# Miscellaneous Notices

**Published:** 1856-04

**Authors:** 


### MISCELLANEOUS NOTICES.

*Resignation.*—It will be seen by the following letters and resolutions that Prof. A. A. Blandy has resigned the chair of Practical Dentistry in the Baltimore College of Dental Surgery.—Eds.

*Baltimore, March 5th,* 1856.

Prof. P. H. Austen,

Dear Sir:—Permit me, through you, as the official organ of the Faculty of the Baltimore College of Dental Surgery, to tender my resignation of the chair of Practical Dentistry in said institution. The time required to attend to its duties, from 9 A. M. to 12 o'clock, five months of the year, is more than I can spare from the practice of my profession, and hence I feel myself called upon to sever the pleasant relation which has so long existed between us.

Wishing the Faculty abundant success in their arduous and praiseworthy labors. I have the honor to be very sincerely and truly,

Yours, ALFRED A. BLANDY.

*Baltimore, March 8th,* 1856.

Dr. A. A. Blandy,

Dear Sir:—In accepting the resignation tendered in your letter of the 5th inst. the Faculty request me to express to you their sincere thanks for the warm interest you have ever manifested in the welfare of the College, and for the sacrifices made by you in furtherance of its success. We regret that any circumstances should have determined you to withdraw from official relations, that have been uniformly marked by harmony and friendly feeling. Permit us to hope, that in the midst of your increasing professional cares, you will preserve an undiminished interest in the future welfare of an institution, whose Faculty desire here to acknowledge her many obligations to you.

I have the honor to be your most obedient servent,

P. H. AUSTEN, *Dean of the Faculty.*

At a meeting held by the graduating class of the "Baltimore College of Dental Surgery," Feb. 22nd, 1856, the following proceedings were had:

Geo. C. Lewis was called to the chair and J. W. Whitmore appointed secretary; when Mr. B. H. Padgett briefly explained the object of the meeting, and offered the following preamble and resolutions, which were afterwards adopted by the class:

Whereas, we have learned with regret of the withdrawal of Dr. A. A. Blandy from the professorship of Dental Practice in the Baltimore Dental College, therefore, be it

*Resolved,* That his efforts to sustain the reputation of the Infirmary, and the impartial and instructive labors he has bestowed in this department, met with our hearty approval.

*Resolved,* That in retiring from this worthy position, which he has so ably filled, he has our warmest sympathies and best wishes.

*Resolved,* That a copy of these proceedings be signed by the chairman and secretary, and transmitted to Dr. Blandy, and also one to the Editors of the American Journal of Dental Science.

On motion, the meeting adjourned "sine die."

GEO. C. LEWIS, *Chairman.*

J. W. Whitmore, *Secretary.*

*Appointment.*—The friends of the Baltimore College of Dental Surgery, will be gratified to learn that the chair of Dental Practice, vacated by the resignation of Prof. Blandy, has been filled by the appointment of Dr. Maynard, of Washington city—a gentleman well known as one of the most distinguished practitioners in the profession.

It will also be seen by the advertisement of the Session for 1856-'7, that the Faculty have created a Lectureship of *Microscopic and Comparative Dental Anatomy,* which has been filled by the appointment of Dr. Christopher Johnston—a gentleman eminently qualified to give a thorough course of instruction in these highly interesting departments of science. By this last appointment, the curiculum in the Baltimore College, has been somewhat extended and rendered more complete.

*Baltimore College of Dental Surgery.*—The sixteenth annual commencement of the Baltimore College of Dental Surgery, was held in the Assembly Rooms of the College, on the evening of the 4th of March. The exercises were opened with prayer by the Rev. Dr. McCabe, after which was read by the Dean, the following list of graduates, with the subjects of theses.

Asa Holmes Balderston, Baltimore, Md., Anatomy and Physiology of the Human Economy.
Samuel A. Bruce, Farmville, Va., Odontrophy.
Thomas J. Corpening, Morganton, N. C., Effects of the Retention of Carious Teeth.
Richard C. Cyphers, Woodbridge, N. J., Vitality.
William F. Edington, Dundee, N. Y., Dental Caries and its Treatment.
William H. Hoopes, Baltimore, Md., Dental Physiology.
Charles M. King, Philadelphia, Pa., Arsenic.
Joseph M. Lauck, Washington, D. C., Extirpation of Dental Pulp.
George C. Lewis, Raleigh, N. C., Abscess.
George W. Neidich, Carlisle, Pa,, Irregularities of the Dental Arch.
Berrymon H. Padgett, Hicksville, N. C., Preservation of the Teeth.
Christopher W. Reed, Wincheter, Va., Dental Hygiene.
R. Bruce Reynolds, Philadelphia, Pa., Mercurial Stomatitis.
Edward W. Swentzel, Lancaster, Pa., Inflammation of the Mouth.
Thomas O. Walton, Annapolis, Md., Saliva.
Elias Weiler, Belleville, Pa., The Teeth.
John W. Whitmore, M. D., Houston, Miss., Difference between Bone and Teeth.

The Graduates then received their Diplomas from the President, and were afterwards addressed by Dr. Solyman Brown, of New York City. In concluding this address, which will be found in the present number of the Journal, Dr. Brown offered three knotty problems for solution: viz. 1st, "Why does the melted wax of the common honey bee, when allowed to cool slowly in a thin stratum, assume geometrical forms of the same average size and shape of the alveoli of the comb from which the wax is derived." 2nd, "Why do all the colors of the solar bow, or prismatic spectrum which is simply an analysis of the sunbeam, from black to white, namely, red, yellow and blue, with all their shades and combinations, enter as pigments into the human hair, blue only, excepted?" 3rd, "Why did the healing art, including medicine and dentistry, have, in darker ages of the world, a greater proportion of *quack* practitioners than any other art or science?" After the conclusion of the address, Prof. Thomas E. Bond, at the suggestion of one of the faculty, arose and gave the following impromptu solutions:

Once upon a time, when, as in the present instance, a large audience had listened to an address by a remarkably eloquent speaker, a gentleman arose and observed that, ***inasmuch as the audience seemed reluctant to disperse he would make a speech.*** A most sagacious resolution, as certainly it was the readiest way to accomplish the dispersion. In like manner and perhaps for similar reasons, I have been requested to add a few words, (by way of putting you in a humor to go home.) In one respect, I have the advantage of the gentleman who has just addressed you—I am not restricted to '20 minutes and no poetry;' indeed, if I were, I think I might transgress and yet follow in the path of safe precedent. My good friend, the doctor, contrived to occupy a good deal more than the time prescribed, (and I am glad that he did,) and I am by no means sure that he did not give you some poetry. If Maud and Hiawatha be poetry, I am rather inclined to think we had some to-night. For modern poetry seems to have become independent of versification. Dr. Brown professed to be an 'old fogy,' and gave you a philosophical definition of the term. If this definition be admitted, I confess that we have had a brilliant display of 'old fogyism' to-night. The glorious sun never seems so resplendant, as when bursting through a mass of clouds, he throws his radiance upon the darkness behind him, and projects his brilliant rays into the clear atmosphere beyond; and certainly, *Old Sol* never exhibited greater brilliancy than he did to-night, when emerging from the fogs of immature life, he threw a flood of light upon the obscure past and into the brighter future.

In taking leave of you, permit me to avail myself of certain enigmas which my worthy friend threw out for solution. I think that he took us at unfair advantage, because in the relation we occupy to you, it is, of course, impossible for us to admit ignorance of anything, and really, a few minutes are scarcely sufficient for the making or solving riddles like these. Yet I must venture. Well, the first was the query about wax. 'Why does melted wax, in cooling, show a tendency to assume the form of its original comb-cell?' In the first place, I am forcibly reminded of a story about a problem, that was said to have been submitted to a learned body, to ascertain why a dead fish weighed more than a living one. After a good deal of scientific discussion, somebody suggested that it might be well to verify the fact before they should explain it, and on weighing, no difference was found to be observable. But admitting the wax story, which I do not vouch for, how can we explain it? Obviously it seems to me, the conduct of the wax is the result of habit. And here let me urge upon you an important moral. If wax, of all things most pliable and plastic, and easily modified in form; if this most tractable of substances, even after having been subjected to the fiery ordeal of a furnace, and reduced to entire change of form; if even wax, from force of habit, nevertheless, gets back to its old condition, what suppose you is likely to be the force of habit in the human mind; so self-willed, so subject to the power of memory and association—so intractable as it is? Now here is the mythical wisdom, which I suppose our learned friend has wrapped up in the wax. At any rate, it is an instructive illustration, and an 'old fogy' myself, I can from experience, assure you that the bad habits inadvertently or foolishly formed in youth, are the great difficulties—the inconsolable regrets of later life.

The next riddle given us for solution, was to explain the parsimony of nature in the use of 'blue' in the coloring of man. The reason of this is very plain. Obviously, nature never intended man to be *'blue.'* There is no use in being *'blue'*—no effect comes of it—no end is accomplished by it. Better have anything else than have the 'blues.' The man who despondingly sits down to ponder over evils and discount coming trouble, is of very little use to himself or others. We are not providences but there is a great and wise Providence over us. With this trust in our hearts, we may fearlessly and hopefully meet the future. Cheerfulness is necessary to successful effort. Nature did well to put so little *blue in us.* Some of us have too much, notwithstanding. So here is another myth unravelled, and a pretty myth it is.

The third problem, we can only make a feeble attempt to solve, and I must help cut the solution by a story.

Some years ago an atheist in this city—for there are such people in the world, who do not know how they came here, or what they are for, or who is who or what is what—well one of them came to his senses and determined to renounce his unbelief publicly. After doing so, he observed that probably his old friends might like to know which of the Christian sects he intended to join, and he had only to say, that he intended to join the one in which he should find the most hypocrites: for said he, men do not counterfeit base coin—upon this principle perhaps it is that there are so many counterfeiters of medicine. The purity of its truth tempts to dishonest pretension.

And now, gentlemen, you are going forth to practice your profession, and I am glad to believe that you will start in life with more than ordinary prospects for success. Remember however the remarks about "success" made by the orator of the evening. Wealth is not success. We have abundant evidence of this. We see many men who have spent years in destroying their sensibilities, in order to accumulate wealth: but surely these men are poor examples of a successful life. Do not fail as they have done. The word "failure" when applied to the result of human life is an awful, a terrible word. To fail in the use of a preliminary and probationary life: to fail in view of eternal results: to fail irremediably—these are fearful thoughts: yet men all around you are so failing, and so will you, unless you at once form right views of the end to be attained and set yourselves resolutely, humbly, and cheerfully to accomplish them.

*Operations performed in the Infirmary of the Baltimore College of Dental Surgery, during session* 1855-'56.

*Medical and Surgical Department.*

| | |
|---|---|
| Extraction of teeth | 2430 |
| Fillings (crystal gold, gold foil and tin foil) | 965 |
| Do. over exposed nerve | 9 |
| Do. to extremity of fang | 8 |
| Superficial caries removed | 98 |
| Cases of removal of tartar | 35 |
| Do. do. diseased gums | 30 |
| Do. do. irregularity | 3 |
| Pivot teeth | 15 |
| Disease of maxillary antrum | 1 |
| Fungous tumor of the upper jaw | 1 |
| *Mechanical Department.* | |
| Double sets, 9 | 18 |
| Full upper sets | 19 |
| Full lower sets | 5 |
| Partial upper sets | 18 |
| Partial lower set | 1 |
| Total number of pieces | 61 |

P. H. Austen, *Dean of the Faculty.*

In the above statement no account is taken of experiments made by students in filling teeth *out* of the mouth; or of a very large number of experimental pieces made not to be worn by patients, but to give practice in the various mechanical details.

*Philadelphia College of Dental Surgery.*—The last commencement of the above Institution, was held the 27th of February, at which time the following gentlemen received the degree of *Doctor of Dental Surgery.* The valedictory was delivered by Prof. Flagg.

J. Canning Allen, Jr. Wm. T. Arringeon, James P. Broun, Charles H. Burr, Louis Martin y De Castro, Antonio L. Coöpat, Francis Field, J. Foster Flagg, James E. Garrettson, William Grimes, John W. Hunter, Jose G. Lopez, Samuel Martin, M. D.,

A. F. McLain, Robert L. McClellan, Charles Neil, M. D., Henry B. Parry, Alan W. Read, W. Bartling Robbins, R. Woodward Robinson, John Z. Stanger, James K. Whiteside.

We copy the following from the New York Dental Recorder: the editor of which was present on the occasion.

The following is a record of the operations performed in the infirmary:—

*Demonstrator's Reports.—Session of* 1855-'6.

*Operative Department.*

| | |
|---|---|
| Fillings | 562 |
| Treatment of "nerve," (cases) | 84 |
| Extraction of teeth and roots | 723 |
| Superficial caries | 2 |
| Removal of salivary calculus, (cases) | 27 |
| Pivot teeth set | 7 |
| Total operations | 1405 |

Louis Jack, *Demonstrator.*

*Mechanical Department.*

| | |
|---|---|
| Entire sets of teeth | 3 |
| Partial sets | 16 |
| Total of teeth inserted | 163 |

W. Calvert, *Demonstrator.*

R. Arthur, *Dean of the Faculty.*

It will at once be seen that in Dental Colleges students have opportunities of witnessing and performing many more practical operations on the teeth, in the course of a few weeks, than they could possibly hope to meet with by remaining years with the most competent teacher, even were he backed by the most complaisant of patients.

During the commencement exercises, an incident occurred that struck us as being something novel in the history of Dental Surgery.

The President (we presume) of the Board of Trustees or Corporators read the authority for conferring the degrees, and handed the diplomas to the Graduates. After they had all been conferred, and the Graduates had resumed their seats, the President announced that the Honorary Degree had been conferred upon two persons, stating their names and residences, which we have forgotten, (further than that they lived in that small part of the State of Camden and Amboy known as New Jersey.) The peculiarity of this procedure consisted in the neglect, on the part of the President, to state that these Honorary Degrees had been conferred upon the said parties by the Board, and against the wishes of the Faculty, who had, we understand, entered their protest against any such action.

We learn that among the board of corporators there is but one dentist to be found. The faculty of the colleges are, on the contrary, all practical and practicing dentists. They are considered as the responsible parties by the profession, and are unquestionably the only persons connected with the institution who can be considered as judges of professional ability. Looking at the question in this light, we must confess that the action of the board of corporators argues for them an amount of presumption and a want of principle that might be expected among politicians, but that no one would look for among the trustees of a scientific or literary institution. Of course, no gentleman could accept a degree conferred under such circumstances.

After the commencement exercises were over, the guests, students, &c., adjourned to Parkinson's, where a collation was disposed of in a manner satisfactory to all parties. A number of speeches were made, and the evening passed of very pleasantly.

Prof. Arthur took occasion, in a very frank and manly address, to protest publicly against the procedure of the board of corporators alluded to above. His remarks were received with unqualified applause and an enthusiasm which evinced most satisfactorily that the sympathies of the dental profession were on the side of the faculty.

We have since learned that at a special faculty meeting, held on Monday evening last, the faculty resigned. This seems a most summary method of blotting a very flourishing institution out of existence; but we do not see that any other course was open to them.

If the board of trustees had the power and inclination to grant degrees against the wishes of the faculty, they would soon begin to refuse degrees to those whom the faculty thought were entitled to them. The truth of the matter is, that the odium of the ruin of the school must rest upon the heads of those who by their most unwarrantable and unprincipled behavior, have compelled the faculty to make choice between their self-respect and their position in the college. Their action in the matter merits the thanks of the dental profession.

---

*American Medical Association.*—The ninth annual meeting of the American Medical Association will be held in the *City of Detroit,* Michigan, on Tuesday, May 6th, 1856.

The secretaries of all societies and other bodies entitled to representation in the association, are requested to forward to the undersigned correct lists of their respective delegations, *as soon as they may be appointed;* and it is *earnestly* desired by the committee of arrangements, that the appointments be made at as early a period as possible.

The following extracts are from article 2d of the constitution:

"Each local society shall have the privilege of sending to the association one delegate for every ten of its regular resident members, and one for every additional fraction of more than half this number.

"The faculty of every regularly constituted medical college or chartered school of medicine, shall have the privilege of sending two delegates. The professional staff of every chartered or municipal hospital, containing a hundred patients or more, shall have the privilege of sending two delegates; and every other permanently organized medical institution, of good standing, shall have the privilege of sending one delegate.

"Delegates, representing the medical staff of the United States army and navy shall be appointed by the chiefs of the army and navy medical bureau. The number of delegates so appointed shall be four from the army medical officers, and an equal number from the navy medical officers."

The latter clause, in relation to delegates from the army and navy, was adopted as an amendment to the constitution, at the meeting of the association held in New York, in May, 1853.

*** Medical Journals, &c., please copy.

William Brodie, M. D., Detroit, Mich.,
*One of the Secretaries.*

---

***Aluminium or Aluminum.***—The senior editor acknowledges the kindness of Messrs. Charles Abbey & Sons, from whom he has received a few sheets of foil prepared from this newly discovered metal. He has been enabled to experiment sufficiently to convince him that a good filling can never be made with it; and that in this department of dentistry, it can never compete with gold. In the department of dental mechanics, however, we think it may be destined to play an important part: and we await with much interest the result of certain experiments to be made by Professor Austen, who has ordered a supply of the metal from Paris.

Our readers will find in the present number a very interesting article upon aluminium. We also subjoin the letter of Messrs. Abbey & Sons; it presents, very clearly, one of the properties that unfit it for use as foil.

*Philadelphia, Feb.* 23, 1856.

Dr. C. A. Harris, Baltimore, Md.

Dear Sir:—Enclosed please find a sample of foil made of aluminium. The attention of scientific and practical men has been strongly directed, within the last two or three months, to this metal, by recent discoveries by certain French chemists, of increased facilities for producing the metal, and the confident expectations held forth, that it will be still more abundantly produced by further discoveries. It is claimed for aluminium that (when it shall become more abundant) it will be a substitute for gold and silver in many of the trades, &c., in which those costly metals are now used, and it has been suggested that it will prove well adapted to the uses of the dentist. With a view to give some of the inquiring, thinking men of the profession an opportunity of testing how far it is adapted to their use for "filling cavities" in teeth, we have embraced the first opportunity offered of procuring a specimen and working it into foil for distribution. We were enabled to obtain but a small quantity, our entire lot for distribution consisting of less than 100 leaves, hence we send you no larger sample than the enclosed half dozen. We find the metal tolerably kindly, a little disposed to be harsh, or "sort of stiff," but still ductile, and to a considerable degree tough, color maintained without change, throughout whole process of reduction from original ingot to foil, inclined to be scaly, as you may see, caused by its lacking that quality in gold of "uniting," or as we may express it, "welding" together during "beating," when it has become broken or seems opened in it by carelessness or accident in that process. In gold the seams or fracture may be joined together ("closed up" is the technical) under the hammer so as to be unseen, but the aluminium does not unite, the edges of the fracture will overlap and continue to spread under the hammer in two thicknesses. More explicitly, two sheets of gold foil laid one upon the other may be inseparably united by "beating;" but two of aluminium will remain two, all the time. The enclosed foil is *thicker* probably, than what you ordinarily use, but the average weight of each sheet is about 2½ grs. The specific gravity is represented to us as about one fourth that of silver. Each sheet has been annealed to a dull red heat, melting easily beyond that. When it shall be well ascertained that aluminium undergoes or causes no change in the mouth, we think it will prove a valuable substitute for gold and silver plate in mechanical dentistry.

Yours, respectfully, CHARLES ABBEY & SONS.

*Baltimore Dental Depot.*—Mr. Snowden, of Baltimore, has recently opened a depot, corner of Market and Eutaw streets, for the accommodation of the dentists of Baltimore, as well as those visiting the city for the purpose of supplying themselves with porcelain teeth, gold, platina, instruments, &c. In short he proposes to keep all the materials used by the profession, and will furnish them on as reasonable terms as they can be procured in Philadelphia or New York. Having obtained the agency for the sale of the teeth made by the New York Teeth Manufacturing Company, he is supplied with a large and beautiful assortment, which for natural appearance and strength are equal to any we have seen. He also keeps the various patterns of files manufactured by this company, which in some respects are superior to any we have ever used.

The want of a dental depot in Baltimore has been long felt, and now one is established, we trust it will be sustained by the profession of the city.

*New York Teeth Manufacturing Company.*—The senior editor is indebted to Mr. Crowell, one of the actuaries of this enterprising company, for several samples of their dental files, which are equal in quality to any which he ever had from Stubb's establishment, of England, while the patterns are more numerous and better adapted to operations on the teeth. He feels confident they will receive the approbation of all who use them. He has also had an opportunity of examining some of their plate teeth, which are very beautiful, and admirably adapted to the various methods of mounting.

*Dentists Soldering Lamp.*—Mr. T. J. Haskell, dentist, of Philadelphia, has recently constructed a soldering lamp, possessing several advantages over those usually employed for the purpose. It will hold about a pint of alcohol; upon the top a piece of slate is let in for grinding borax, as well as a place for the borax and solder. To the back part of the lamp there is a cup for water and a match box at the side. Near the spout for the wick is, what we presume is intended for a ventilator or escape pipe for any steam which may be generated inside while soldering. We do not, however, perceive that this can be of any particular advantage, as this will pass off through the wick-spout as generated; besides, it seems to us, though we may be mistaken, never having used the lamp, that the flame might be communicated to the alcohol within through this tube. This, however, might be easily prevented by closing the tube. There is also a place on the side for pouring the alcohol into the lamp.

*Medico-Legal Importance of Cicatrices.*—By Schneider.—The appearance of cicatrices is important in medical jurisprudence, inasmuch as it depends on the nature of the injury received, and on the sort of instrument by which it was inflicted.

*Cicatrices after incised wounds,* when these have been properly treated and there has been no deep-seated suppuration, generally present a more or less rectilinear form. But the elasticity aud stretching of the skin, the convexity of the subjacent parts, and the softness of the cellular tissue, may change a rectilinear cicatrix into an elliptic, or even into a circular one. *Cicatrices from stabbing instruments* are often of a triangular form. Those from *gunshot wounds* are generally of a rounded or grooved character. If the shot has been received from some distance, there is a considerable mark (*scheibe*) with depression of the skin in the centre, and puckering at the margins; if the shot had been fired close at hand, the cicatrix is deeper, its edges are irregular, and it is of a bluish color. In wounds of this description, also, the point of entrance of the ball is shown by a depressed, and the point of its exit by an elevated cicatrix. *Bruises,* when unattended with lesion of the integuments, leave no cicatrix; but when occurring with this complication, they leave cicatrices resembling those caused by incised wounds, or injuries accompanied with loss of substance. ***Bites of mad animals*** are distinguished by no peculiarity of scar. ***Burns*** of the first, or minor grade, leave no mark; those of the second grade occasion scars like those of blisters; those of the third grade cause white, glistening, and tolerably smooth cicatrices; and those of the fourth, or most severe grade, leave dense, hard, irregular, contracted cicatrices, with abnormal union of approximated surfaces, and very great retraction of the cellular tissue. ***Fluid cauterants*** give rise to superficial, and ***solid cauterants*** to deep circumscribed cicatrices, with contracted centres, and these may in certain cases be variously colored by the chemical nature of the irritant substance. ***Scrofulous ulcers*** leave cicatrices which are irregular and furrowed, with smooth deep depressions, and surrounded by hard uneven margins. The ***scorbutic cicatrix*** is dark bluish-red in color, soft to the touch, somewhat elevated, and rather painful; in course of time it becomes flatter, of a reddish brown color, approaching to green in the centre, and very thin and easily injured. ***Arthritic scars*** are of a reddish brown, a bluish, or an ash-gray color; they are studded with elevations and depressions; while their circumferences are dark brown, varicose, and often inflamed and erysipelatous. ***Herpetic cicatrices*** are wider spread, irregular, superficial, and of a dirty reddish-brown color; in the centre they are often of the same color as the old skin, and they gradually merge into the surrounding integuments. ***Syphilitic cicatrices*** are characterized by great loss of substance; they approximate the lips of the deep ulcers before their granulations have had time to reach the surface. ***Glandular cicatrices*** are irregularly tumefied; generally deep, hardened, and of a reddish-brown color. The cicatrices of ***true smallpox*** are flat and irregular, with indented margins, giving to the surface the appearance of a citron; while those of ***pseudo-variola*** have smooth even borders, and have hairs growing in them.

As regards the ***age of a cicatrix,*** we must be guided by its color, size, and thickness, in forming of our opinion. The fainter its color, the larger its extent, and the greater its sensibility and tenderness, the more recent is its formation, and the more imperfect is its organization. As its age increases it grows smaller, thicker, more shining, and less sensitive.—***Monthly Journ. Med. Sci.***, Oct. 1854, from ***Prag. Viertaljahrs.*** Bd. ii, 1854.

***A Gold Filling Removed from the Neck.***—A lady in Tennessee, had a gold filling, about the size of a squirrel shot, recently removed from the side of her neck, immediately beneath the angle of the jaw. She was ignorant of the manner in which it got there, but it probably escaped from one of her teeth while eating and lodged in the fauces or a fold of the mucous membrane of the lower part of the mouth, and from thence made its way to the place from which it was removed.

*Triumph in Dentistry.*—A professional friend writing to the senior editor, states that a dentist of the state of New York, has attained to such perfection in the use of crystalline gold, that he can build on to the roots of a firmly articulated molar, an entire crown, uniting it to them so firmly that the tooth may afterwards be extracted by the application of forceps or a key to the artificial crown. The next time we hear from our correspondent, we shall expect to receive the gratifying intelligence that some of our progressive brethren at the north have succeeded in replacing with gold, roots as well as crowns, of recently extracted molars, to answer a better purpose than masticators supplied by nature.

*Killing two Birds with one Stone.*—A new method of economising time in operating on the teeth was mentioned to us a few months since by a professional friend of New Orleans. A gentleman, suffering from pain in a tooth, applied to a dentist in the south for relief, and not being willing to submit to the loss of the organ, the decomposed portions were partially removed, and arsenious acid applied to the bottom of the cavity on a bit of raw cotton. This done, the operator at once proceeded to fill the tooth with gold, assuring his patient that the pain would entirely cease in a few hours. The prediction proving correct, the gentleman supposed he had been peculiarly fortunate in securing the services of a most skillful practitioner, nor was it until several weeks had elapsed, when his tooth again become troublesome, that he became aware of the malpractice to which he had been subjected. He now applied to the professionalgentleman, to whom we are indebted for the particulars of the case, who, on removing the filling discovered the imposition that had been practiced upon his patient.

*A Valuable Bucket.*—Among the many modes of making money here, none, I think, surpasses the following: A surgeon told me he went one day into the tent of a brother medicus, on the Bendingo, just as a patient was going out, "I have been stopping a tooth," said the surgeon. "Do you get good cement here?" inquired my friend. "Admirable! I saw an old gutta percha bucket selling in a lot of tools one day at an auction. I bought the lot for the sake of the bucket, which cost me five shillings. I have already stopped some hundreds of teeth with the gutta percha at a guinea each, and shall, no doubt, stop thousands with it before the old bucket is used up. It is a fortune to me. My name is up for an unrivalled dentist, and they come to me from far and near."—*Two Years in Victoria.*

